# Prevalence and Genetic Characterization of *Giardia duodenalis* and *Blastocystis* spp. in Black Goats in Shanxi Province, North China: From a Public Health Perspective

**DOI:** 10.3390/ani14121808

**Published:** 2024-06-17

**Authors:** Han-Dan Xiao, Nan Su, Ze-Dong Zhang, Ling-Ling Dai, Jun-Lin Luo, Xing-Quan Zhu, Shi-Chen Xie, Wen-Wei Gao

**Affiliations:** 1 Laboratory of Parasitic Diseases, College of Veterinary Medicine, Shanxi Agricultural University, Taigu, Jinzhong 030801, China; 15166873600@163.com (H.-D.X.); sunan1228@163.com (N.S.); zzd18203541696@163.com (Z.-D.Z.); 13081822906@163.com (L.-L.D.); l1076091241@163.com (J.-L.L.); xingquanzhu1@hotmail.com (X.-Q.Z.); xieshichen221@163.com (S.-C.X.); 2 Key Laboratory of Veterinary Public Health of Higher Education of Yunnan Province, College of Veterinary Medicine, Yunnan Agricultural University, Kunming 650201, China

**Keywords:** *Blastocystis* spp., *Giardia duodenalis*, black goat, prevalence, multilocus sequence typing, zoonotic parasites, Shanxi Province

## Abstract

**Simple Summary:**

*Blastocystis* spp. and *Giardia duodenalis* are two prevalent intestinal parasites with a worldwide distribution that can infect humans and animals, resulting in significant public health concerns and economic losses. For goats, infection with the two parasites can cause symptoms such as diarrhea, which is not conducive to the development of stockbreeding. Shanxi Province is one of the largest goats breeding provinces in China. However, the prevalence of *Blastocystis* spp. and *G. duodenalis* in black goats in Shanxi Province remains unknown. Thus, 1200 fecal samples of black goats were collected in five representative geographical locations in Shanxi Province to examine the presence and genotypes of *G. duodenalis* and *Blastocystis* spp. by using a molecular approach. The results showed that the total infection rates of *G. duodenalis* and *Blastocystis* spp. were 7.5% and 3.5%, respectively. The presence and genotypes of *G. duodenalis* were determined based on three established loci (*tpi*, *bg*, and *gdh*). Among the detected assemblages B and E of *G. duodenalis*, the most prevalent assemblage was E in black goats in the five study areas. One novel MLG (MLG-E12) was identified by multilocus genotypes (MLGs) analysis. Through DNA sequence analysis, four subtypes of *Blastocystis* spp. were found in black goats, namely ST5, ST10, ST14, and ST30, among which ST10 was the dominant subtype in this study. This is the first report of *Blastocystis* spp. and *G. duodenalis* infection in black goats in Shanxi Province, which not only enhances our understanding of the genetic diversity of *Blastocystis* spp. and *G. duodenalis* in black goats in China but also provides essential baseline data for the prevention and control of *Blastocystis* spp. and *G. duodenalis* infection in black goats in the study areas.

**Abstract:**

*Blastocystis* spp. and *Giardia duodenalis* are two prevalent zoonotic intestinal parasites that can cause severe diarrhea and intestinal diseases in humans and many animals. Black goat (*Capra hircus*) farming is increasingly important in China due to the remarkable adaptability, high reproductive performance, rapid growth rate, and significant economic value of black goats. A number of studies have indicated that black goats are the potential reservoir of multiple zoonotic protozoans in China; however, the prevalence and zoonotic status of *G. duodenalis* and *Blastocystis* spp. in black goats in Shanxi Province is still unknown. Thus, a total of 1200 fecal samples of black goats were collected from several representative regions at different altitudes in Shanxi Province and were examined for the presence and genotypes of *G. duodenallis* and *Blastocystis* spp. by amplifying the beta-giardin (*bg*), glutamate dehydrogenase (*gdh*), and triosephosphate isomerase (*tpi*) loci of *G. duodenalis* and SSU rRNA of *Blastocystis* spp. using PCR and sequence analysis methods, respectively. The overall prevalence of *G. duodenalis* and *Blastocystis* spp. in black goats in Shanxi Province were 7.5% and 3.5%, respectively. Two assemblages (B and E) of *G. duodenalis* and four subtypes (ST5, ST10, ST14, and ST30) of *Blastocystis* spp. were identified, with assemblage E and ST10 as the prevalent genotype and subtype in black goats, respectively. One novel multilocus genotype (MLG) was identified in MLG-E and was designated as MLG-E12. For both *G. duodenalis* and *Blastocystis* spp., the prevalence was significantly related to the region and age groups (*p* < 0.05). This is the first report on the prevalence of *G. duodenalis* and *Blastocystis* spp. in black goats in Shanxi Province. These results not only provide baseline data for the prevention and control of both parasites in black goats in Shanxi Province, but also enhance our understanding of the genetic composition and zoonotic potential of these two parasites.

## 1. Introduction

Giardiasis and blastocystosis are two common gastrointestinal diseases in both humans and most animals caused by protozoal parasites known as *Giardia* and *Blastocystis* spp., respectively [1,2]. Healthy humans and animals with competent immunity are usually asymptomatic [3]. However, immunocompromised individuals ingest the cysts of *Giardia* or *Blastocyst*, which are contained in the food or water via the fecal–oral route, resulting in stunted growth and gastrointestinal disturbances, such as diarrhea [4,5].

The Giardia genus is considered to have eight valid species, including *Giardia microti*, *Giardia cricetidarum*, *Giardia muris*, *Giardia ardeae*, *Giardia psittaci*, *Giardia agilis*, *Giardia peramelis*, and *Giardia duodenalis*. Of them, *G. duodenalis* (synonmy *G. intestinalis* and *G. lamblia*) is the only species of *Giardia* that infects humans [6,7,8,9]. In early investigations, previously reported *G. duodenalis* were divided into eight assemblages/genotypes (A–H), in which A and B belonged to the zoonotic group [10,11], and C–H were considered as host-specific assemblages: C and D for canines, E for artiodactyls, F for felines, G for rodents, and H for marine mammals [12]. In the last decade, assemblages C, D, E, and F were also detected or isolated from human stool specimens; however, the zoonotic role of these assemblages in humans was still unknown [13,14]. In recent years, multilocus genotyping (MLG) of the beta-giardin (*bg*), glutamate dehydrogenase (*gdh*), and triose phosphate isomerase (*tpi*) loci has increasingly been used to characterize *G. duodenalis* infection in humans and animals [15]. In developing countries, the prevalence of *G. duodenalis* infection ranges from 8.0% to 30.0% in children and from 1.5% to 42.2% in sheep and goats [16]. In addition, assemblage E has been found in humans infected with *G. duodenalis* in Europe and Egypt [16]. In 2022, *G. duodenalis* was detected in feces samples from western chimpanzees and individuals with close contact in Comoé National Park, Côte d’Ivoire, which indicates the risk of zoonosis [17]. Additionally, assemblage E was the most frequently detected assemblage in goats in the world, followed by assemblages A and B [18]. In China, MLG-E is often identified in goats; however, MLG-A has also been detected in Yunnan Province, southwestern China [19].

Regarding *Blastocystis* spp., a total of 28 distinct lineages have been identified and recognized based on nucleotide polymorphisms within the small subunit ribosomal RNA genes (SSU rRNA) of *Blastocystis* spp. and designated as subtypes ST1 to ST17, ST21, ST23-ST29, and ST30-ST32; with ST1-ST9 and ST12 commonly detected in humans and in other mammals [20,21,22]. More evidence indicated that ST1 to ST4 contributed to more than 90.0% of human infection cases, in which ST1 and ST3 have the strongest pathogenicity, and ST3 was commonly detected in school children [23,24]. Worldwide, the *Blastocystis* spp. prevalence in humans was documented in Europe (22.0% to 56.0%) and African and Asian countries (37.0% to 100.0%) [25,26]. Moreover, the global prevalence of *Blastocystis* spp. in goats was 20.5%, with ST10 being the predominant subtype [22].

China is one of the most important agricultural countries in the world and has been the country with the largest number of goats since the late 1980s [27]. Shanxi Province is located inland of the mid-latitude zone of North China and has a temperate semi-humid and semi-arid continental monsoon climate. Black goats (*Capra hircus*), as an endemic goat breed, are extensively bred in many provinces of China, including Shanxi Province, due to their exceptional meat production performance and favorable reproductive capabilities [28,29]. In China, the highest prevalence of *G. duodenalis* and *Blastocystis* spp. in black goats were reported in Guangdong Province (27.5%, 56/226) and Yunnan Province (41.3%, 375/907), respectively [29,30]. Moreover, MLG was detected in black goats from Yunnan Province [31]. So far, there have been no reports regarding the prevalence of *G. duodenalis* and *Blastocystis* spp. in black goats in Shanxi Province. Thus, the objectives of the present study were to explore the prevalence and zoonotic potential of *G. duodenalis* and *Blastocystis* spp. in black goats in Shanxi Province, aiming to provide the baseline data for executing the prevention and control strategy against these two parasites in black goats in Shanxi Province.

## 2. Materials and Methods

### 2.1. Ethics Approval

The experimental procedures of the study were reviewed and approved by the Experimental Animal Ethics Committee of Shanxi Agricultural University (Approval No. SXAU-EAW-2023G.PB.008011172, approved on 11 August 2023). The animals were handled in accordance with good animal practice as defined by the relevant Animal Ethics Procedures and Guidelines of the People’s Republic of China.

### 2.2. Sampling Collection

**Figure 1:**
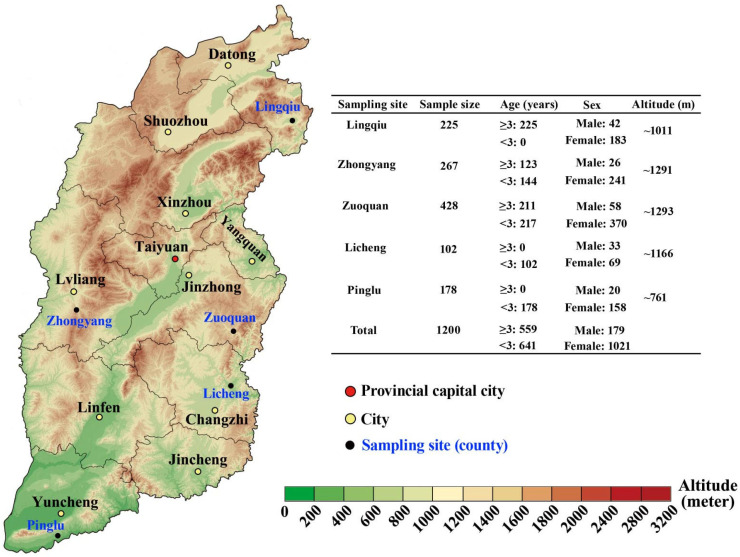
Sampling sites of black goat feces in Shanxi Province, North China.

From October to November 2023, a total of 1200 fresh fecal specimens were collected from black goats without diarrhea in five representative regions of Shanxi Province, including Jinzhong City (*n* = 428), Lvliang City (*n* = 267), Datong City (*n* = 225), Changzhi City (*n* = 102), and Yuncheng City (*n* = 178) (Figure 1). Fresh stool samples were collected from the rectum of each animal and placed in a separate sterile polyethylene (PE) glove, isolated in time to prevent contamination. At the same time, detailed information (region, sex, and age) for each sample was recorded. As shown in Figure 1, all samples were divided into 2 age groups (older goats ≥3 years old and younger goats <3 years old), 2 sex groups (male and female), and 2 altitude groups (>1 km and <1 km). Subsequently, the collected specimens were transported to the Laboratory of Parasitic Diseases, College of Veterinary Medicine, Shanxi Agricultural University, and stored at −20 °C until genomic DNA extraction.

The NOAA’s National Center for Environmental Information (https://gis.ncdc.noaa.gov/maps/ncei/cdo/monthly, accessed on 15 March 2024) was used to extract relevant geoclimatic data (latitude, longitude, elevation, temperature, precipitation, humidity, and climate).

### 2.3. DNA Extraction and PCR Amplification

Following the manufacturer’s instructions, approximately 200 mg of each fecal sample was used to extract the genomic DNA using the E.Z.N.A.^®^ Stool DNA Kit (Omega, Bio-tek Inc., Norcross, GA, USA). According to the previously reported relevant primers (Table S1), the present study amplified the *bg*, *tpi*, and *gdh* loci of *G. duodenalis* and SSU rRNA of *Blastocystis* spp. by PCR method and determined the occurrence and prevalence of both parasites [32,33]. The PCR system consists of 2.5 μL of 10 × PCR Buffer (Mg^2+^ free), 2 μL of dNTPs (25 mM MgCl_2_), 1.25 U of *Ex*-Taq enzyme (Takara, Dalian, China), 1 μL of each primer (20 mM), 2 μL of DNA template, and double distilled water (ddH_2_O) was added to a final volume of 25 μL. PCR amplification procedures referred to previous reports [32,33], and the annealing temperatures are summarized in Table S1. Moreover, 2 μL negative (ddH_2_O) and positive (DNA of *G. duodenalis* or *Blastocystis*) controls were included in each PCR amplification round for detecting the contamination and reliability of results. All PCR products were visualized by 1.5% agarose gels containing ethidium bromide (EB) under UV light, and the positive products were sent to Sangon Biotech Co., Ltd. (Shanghai, China) for bidirectional sequencing.

### 2.4. Sequencing and Phylogenetic Analysis

Each sequence was obtained by overlapping the bidirectional peak map in Chromas V2.6 software. The genotypes and subtypes of *G. duodenalis* and *Blastocystis* spp. were determined by aligning with reported sequences available in the GenBank database using the Base Local Alignment Retrieval tool for nucleotide (BLASTn) on the NCBI website and novel genotypes and subtypes were identified according to the established nomenclature system [34]. Additionally, multilocus genotypes (MLGs) of *G. duodenalis* were used to judge the potential correlation among three genetic markers using the multilocus sequence typing (MLST) tool [31]. Subsequently, the phylogenetic evolutionary trees were separately constructed using identified genotypes or subtypes of *G. duodenalis* and *Blastocystis* spp. using the Neighbor-Joining (NJ) method with 1000 replicates of bootstrap value in Mega 7.0 to further explore the genetic characteristics.

### 2.5. Statistical Analysis

The chi-square (χ2) test was used to explore the correlation between risk factors (region, age, sex, and altitude) and prevalence of the two parasites by SPSS 26.0 software (SPSS Inc., Chicago, IL, USA). Odds ratios (ORs) and their 95% confidence intervals (95%CI) were calculated to assess the strength of the association between prevalence and risk factors. The correlation was considered statistically significant when the *p*-value was less than 0.05.

## 3. Results

### 3.1. The Prevalence of G. duodenalis and Blastocystis spp.

In this study, 90 out of 1200 fecal samples were detected as *G. duodenalis*-positive with an overall *G. duodenalis* prevalence of 7.5%. As shown in Table 1, the presence of *G. duodenalis* was observed in all five sampling sites, with the highest prevalence of *G. duodenalis* in black goats detected in Changzhi City (24.5%, 95% CI: 16.2–32.9). Statistical analysis revealed that significant differences in the prevalence of *G. duodenalis* were observed among region groups (*p* < 0.001), age groups (*p* = 0.005), and altitude groups (*p* = 0.023), but not found in sex groups.

The overall prevalence of *Blastocystis* spp. in the examined black goats in Shanxi Province was 3.5% (42/1200) (Table 1). The highest prevalence of *Blastocystis* spp. was detected in Changzhi City (22.6%, 95% CI: 14.4–30.7), which was significantly higher than that in the other four sampling regions (*p* < 0.001). Likewise, the prevalence of *Blastocystis* spp. between younger goats (4.7%, 30/641) and elder goats (2.2%, 12/559) was statistically significantly different (*p* = 0.017). Notably, the prevalence of *Blastocystis* spp. was not statistically significantly different in relation to altitude and sex factors (*p* > 0.05).

For all the fecal samples investigated in this study, the results showed that both *G. duodenalis* and *Blastocystis* spp. infections were more likely to be found in black goats who were under three years, female, and living in areas with altitude above 1 km. Notably, black goats in Changzhi City had the highest prevalence of the two parasites, significantly higher than that in the other four study areas (Table 1).

### 3.2. Population Genetic Analyses of G. duodenalis

In order to investigate the population genetic structure of *G. duodenalis* in black goats in five study regions, out of 90 *G. duodenalis*-positive samples, 70, 7, and 32 sequences were obtained by further sequencing and analyses at the *bg*, *tpi*, and *gdh* loci, respectively. As shown in Table 2, among the 70 obtained sequences at the *bg* locus, 38 were assemblage B, and 32 belonged to assemblage E. Regarding *tpi* and *gdh* loci, all 7 and 32 sequences were identified as assemblage E. Interestingly, assemblage B was detected in four regions, except Yuncheng City where the altitude was less than 1000 m. In addition, assemblage E was also not detected in black goats in Datong City.

As shown in Table 3, out of the obtained 70 sequences at the *bg* locus, 38 belonged to sub-assemblage BⅢ and 32 represented assemblage E (including 27 known assemblage E35, four known assemblage E1, and one novel assemblage E41). Single nucleotide polymorphisms (SNPs) analysis of assemblage B at the *bg* locus of *G. duodenalis* indicated that 76.3% (29/38) of sequences showed 98.8% similarity to the reported assemblage BⅢ (LC184000) with 4 SNPs, and another 9 sequences exhibited 98.5% to 98.8% similarity with the reference sequence (LC184000) with 4-6 SNPs. Meanwhile, the known sub-assemblage E1 and E35 sequences showed 100.0% similarity to the sequences of assemblage E detected in Tan sheep in the Ningxia Hui Autonomous Region of China with accession number MK610387 (E35) and MK610388 (E1), respectively. Moreover, a novel sub-assemblage E41 was identified in this study with one SNP when referred to the reported assemblage E35 (MK610387). At the *gdh* locus, 30 out of 32 sequences were identical to the known assemblage E34 (MK645786); two sequences showed a 99.8% similarity and were designated as novel sub-assemblage E53 (Table S2). In addition, seven sequences detected at the *tpi* locus showed a 100.0% similarity to the reported assemblage E (EU189333), designated as E33 (Table S3). The representative sequences obtained in the present study were deposited in the GenBank database with the following accession numbers: PP754423 to PP754425, PP754427 to PP754435 for the *bg* gene, PP754419 to PP754420 for the *gdh* gene, and PP754421 for the *tpi* gene.

In this study, six samples were successfully sequenced at all three intra-assemblage variation genetic loci, forming one novel MLG-E12 within the assemblage E (Table S4). This novel MLG was only found in black goats in Changzhi City.

**Figure 2:**
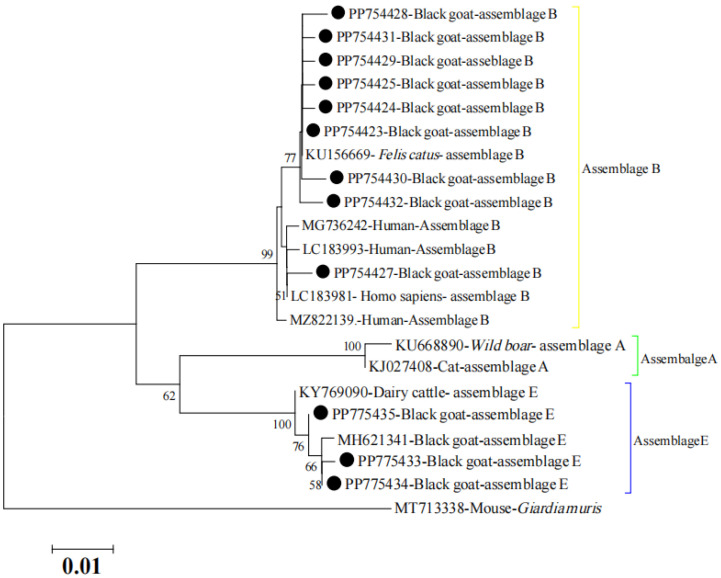
Phylogenetic tree of the obtained sequences (marked with a black circle) in this study and previously reported sequences of *G. duodenalis* based on *bg* loci. The Kimura 2-parameter model method was used with bootstrap evaluation of 1000 replicates. Bootstrap values are shown when >50%.

A phylogenetic tree was conducted to explore the relevance of detected assemblages and sub-assemblages in this study. As shown in Figure 2, the tree revealed that the assemblage E detected in this study consistently clustered with the reported assemblage E and had a close relationship with reported assemblage E in cattle and goats. It is noteworthy that the tree also demonstrated that the novel assemblage B was closely related to the reported assemblage B, which was detected in humans.

### 3.3. Population Genetic Analyses of Blastocystis spp.

As shown in Table 4, sequence analyses indicated that the obtained 42 Blastocystis-positive sequences from the examined black goats were divided into four known subtypes, including ST10 (*n* = 26), ST14 (*n* = 13), ST5 (*n* = 2), and ST30 (*n* = 1). Notably, the prevalent ST10 was detected in black goats in four study areas but not detected in Yuncheng City, and the ST14 was widely observed in Lvliang City, Yuncheng City, Changzhi City, and Datong City, and was the only ST detected in Yuncheng City. In addition, ST30 was only detected in black goats in Jinzhong City.

**Figure 3:**
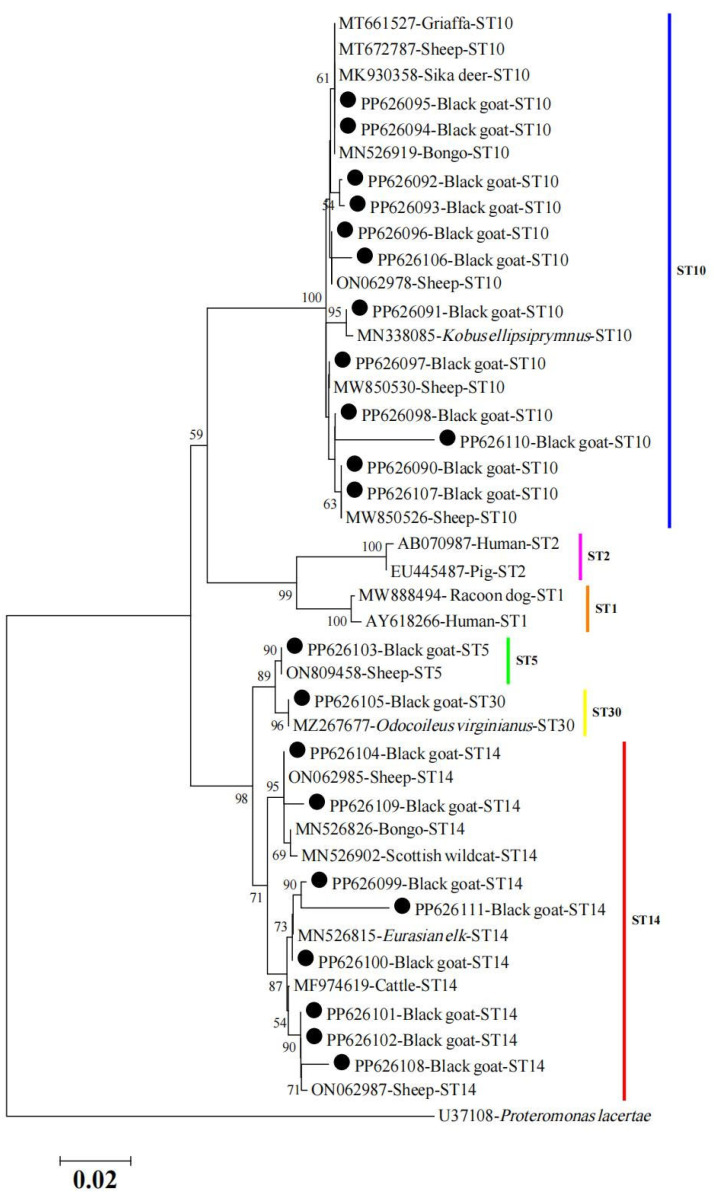
Phylogenetic tree of the obtained sequences in this study (marked with a black circle) and previously reported sequences of *Blastocystis* spp. based on SSU rRNA. The Kimura 2-parameter model method was used with bootstrap evaluation of 1000 replicates. Bootstrap values are shown when >50%.

Moreover, the obtained sequences of *Blastocystis* spp. detected in this study were used to conduct a phylogenetic tree with 21 reported sequences, which were deposited into the GenBank database. As shown in Figure 3, the sequences detected in this study were closely related to and clustered with reported animal-derived STs, and other reported STs were separately positioned in distinct branches (Figure 3). The representative sequences obtained in the present study were deposited in the GenBank database with the following accession numbers: PP626090 to PP626111.

### 3.4. Co-Infection of G. duodenalis and Blastocystis spp. in Black Goats

In this study, among the detected 90 *G. duodenalis*-positive samples and 42 *Blastocystis*-positive samples, nine samples showed the co-infection of both parasites in black goats in Shanxi Province, in which eight samples were collected in Changzhi City (Table 5). Interestingly, among the nine co-infected black goat samples, five of them were successfully identified as *G. duodenalis-*positive at all three loci and formed a novel MLG-E12.

## 4. Discussion

*G. duodenalis* and *Blastocystis* spp. are two common intestinal protozoans that infect humans and many animals, including goats, which can cause diarrhea, weight loss, and reduced feeding efficiency [35]. *Blastocystis* spp. has been reported in over half of the countries across the Asian continent [36]. Furthermore, more than 28.5 million people in China are affected annually by *G. duodenalis*, positioning it as the second most prevalent causative agent for human infectious diarrhea following viral pathogens [37]. To reveal the prevalence and genetic characterization of *G. duodenalis* and *Blastocystis* spp. in black goats in Shanxi Province, the present study detected and characterized both parasites by PCR amplification and sequence analyses.

Overall, the prevalence detected in black goats in Shanxi Province was 7.5% for *G. duodenalis*, with assemblage E being the predominant genotype in the study areas. In the world, *G. duodenalis* was less well studied in goats, with the reported prevalence ranging from 4.0% to 43.5% [13]. Moreover, the *G. duodenalis* prevalence in goats in China ranged from 0 to 46.2% (Table S5). In this study, the prevalence of *G. duodenalis* in black goats in Shanxi Province was higher than that in goats in some countries [38,39] and some provinces of China, such as Yunnan province [19,30,34,40,41,42], but lower than that in goats in some other countries, e.g., Greece and India [43,44,45,46,47,48] and some other provinces of China [29,35,49,50,51,52] (Table S5). These results indicate the importance of goats as hosts for *G. duodenalis* and their potential role in transmitting giardiasis caused by this parasite. Additionally, prevalence differences may be related to various factors, including geographical location, age distribution, sex composition, feeding and management practices, ecological conditions, animal immune statuses, and other contributing factors [7].

Statistical analysis revealed that significant differences in the prevalence of *G. duodenalis* in black goats were observed among region groups, age groups, and altitude groups. The highest *G. duodenalis* prevalence was detected in black goats in Changzhi City and was significantly higher than that in the other four study areas (Table 1). Changzhi City, situated in the southeastern of Shanxi Province, has a warm temperate sub-humid continental monsoon climate characterized by high temperatures and humidity levels. We speculated that the favorable climate and environment may contribute to the prevalence and spread of *G. duodenalis*. Additionally, the prevalence of *G. duodenalis* in black goats decreased as age increased, consistent with previous studies in goats [30]. Generally, younger animals are more likely to acquire the parasites than adults, which have stronger immune systems [41]. At altitude groups, 93.3% of *G. duodenalis*-positive samples were detected in areas above an altitude of 1000 m, which indicated that the altitude factor might play an important role in the transmission of *G. duodenalis*. However, there is no sufficient evidence to explain the correlation between the prevalence of *G. duodenalis* and the altitude.

In this study, *G. duodenalis* assemblages E and B were identified in black goat fecal samples; the former was the main genotype (57.8%, 52/90). The results of this study are similar to those reported in sheep and goats from Henan, Sichuan, Yunnan, Shaanxi, Anhui, and Guangdong Provinces and the Inner Mongolia Autonomous Region of China (Table S5). Numerous reports documented that the close association between humans and animals serves as a basis for the transmission of animal-specific subtypes to humans [53]. Assemblage E has been widely reported in artiodactyls, such as cattle, sheep, pigs, goats, and alpacas, with strong host specificity [35]. Notably, assemblage B was only amplified at *bg* loci, and 79.0% assemblage B (30/38) was detected in Jinzhong City, which is close to the provincial capital city, Taiyuan, where has the largest transportation hub and population in Shanxi Province. High population density and public transportation may be related to the fact that the majority of assemblage B occurred in black goats in Jinzhong City. Up to now, among the five studies reporting the presence of assemblage B in goats, assemblage B was always detected in SSU rRNA and the *tpi* locus, including China [34], India [46], Malaysia [39], and Tanzania [54]. However, in Nigeria, assemblage BI was successfully identified at three loci (*bg*, *gdh*, and *tpi*) from fecal samples of goats for the first time, whereas assemblage BⅢ was only detected at *gdh* and *tpi* loci [55]. Sub-assemblage BIII was a valid zoonotic assemblage of *G. duodenalis*, which has been reported in humans and many animals, including wild animals, domestic animals, and companion animals [13]. *G. duodenalis* was detected in human fecal samples in Heilongjiang Province, and assemblages A and B were distributed in all ages [41].

The multilocus genotyping (MLG) employed in this study has been adopted worldwide as a more reliable method of designating *Giardia* (sub)-assemblages to isolates than single-locus genotyping [32,56]. In this study, three loci of *G. duodenalis* were successfully amplified in six samples and designated as a novel MLG-E12. Previous reports highlighted that assemblage E was detected in humans from different countries, suggesting that assemblage E was a potential zoonotic assemblage [57,58]. However, further epidemiological investigations involving larger sample sizes and diverse geographical regions are necessary to gain deeper insights into the *G. duodenalis* transmission dynamics between animals and humans [12,59].

The prevalence of *Blastocystis* spp. in black goats in Shanxi Province was lower than that in Guangdong Province (33.6%) [29], the Tibet Autonomous Region (8.5%) [60], Shaanxi Province (58.1%) [61] of China, Malaysia (30.9%) [62], Portugal (12.7%) [63], and Libya (10.5%) [64]. In this study, mostly *Blastocystis*-positive samples in black goats were detected from Changzhi City, which exhibited a significant difference compared with the other four investigated regions (*p* < 0.001). Many factors could contribute to these variations, e.g., unique climate conditions and poor feeding and management practices [33]. The prevalence of *Blastocystis* spp. in black goats was observed to be negatively correlated with altitude in the other four areas, except in Changzhi City. With regards to the age groups, there was a statistically significant difference in *Blastocystis* spp. prevalence in the examined black goats (*p* < 0.05). According to a previous study, the age factor is also considered as one of the pivotal factors influencing the transmission of *Blastocystis* spp. among animals, but this point is still controversial [33].

In this study, four subtypes (ST5, ST10, ST14, and ST30) of *Blastocystis* spp. were identified in black goats in Shanxi Province, with the predominant subtype being ST10, consistent with previous studies reported in Asia [36]. ST10 and ST14 were the most prevalent subtypes of *Blastocystis* spp. in sheep and goats in Asia, and have been reported in some provinces of China, such as Guangdong Province [29]. ST10 and ST14 were detected in children in Senegal, suggesting their zoonotic potential and threatening public health [33,65]. Importantly, the present study first reported the occurrence of *Blastocystis* ST30 in black goats in China. A previous report detected ST30 in sheep in China [25]; however, the transmission dynamics and pathogenicity of ST30 are still unknown. ST5, a valid zoonotic subtype of *Blastocystis* spp., has been detected in humans, livestock, and wild animals [20], and was detected in the present study in only two fecal samples.

In this study, a total of nine samples were found to be co-infected with *G. duodenalis* and *Blastocystis* spp. (0.8%, 9/1200). Among these positive samples, eight samples were collected from female black goats aged less than 3 years old in Changzhi City. In fact, poor environmental conditions and chaotic management were observed in farms in Changzhi City, which may be the primary reasons for the occurrence of the highest prevalences of *G. duodenalis* and *Blastocystis* spp. in black goats. Therefore, some sanitation measures are necessary to be taken to control the transmission of two parasites in Changzhi City, including regular cleaning and deworming.

## 5. Conclusions

This study revealed the prevalence and genotypes of *Blastocystis* spp. and *G. duodenalis* in black goats in Shanxi Province for the first time. Subtypes (ST5, ST10, ST14, and ST30) of *Blastocystis* spp. and two assemblages (B and E) of *G. duodenalis* were identified in this study, which suggests the risk of zoonotic transmission. These results provide baseline data for the execution of intervention strategies and measures against *Blastocystis* spp. and *G. duodenalis* in black goats in Shanxi Province.

## Figures and Tables

**Table 1:** Factors associated with the prevalence of *Giardia duodenalis* and *Blastocystis* spp. in black goats in Shanxi Province, North China.

Species	Factor	Category	No. of Positive/Tested	Prevalence % (95% CI)	OR (95% CI)	*p*-Value
*Giardia duodenalis*	Region	Datong	3/225	1.3 (0.2–2.8)	1	<0.001
		Jinzhong	35/428	8.2 (5.6–10.8)	6.6 (2.0–21.7)	
		Lvliang	21/267	7.9 (4.6–11.1)	6.3 (1.9–21.5)	
		Changzhi	25/102	24.5 (16.2–32.9)	24.0 (7.1–81.8)	
		Yuncheng	6/178	3.4 (0.7–6.0)	2.6 (0.6–10.5)	
	Altitude	>1000 m	84/1022	8.2 (6.5–9.9)	2.6 (1.1–6.0)	0.023
		<1000 m	6/178	3.4 (0.7–6.0)	1	
	Age	≥3 years	29/559	5.2 (3.4–7.0)	1	0.005
		<3 years	61/641	9.5 (7.2–11.8)	1.9 (1.2–3.0)	
	Sex	Male	8/179	4.5 (1.4–7.5)	1	0.095
		Female	82/1021	8.0 (6.4–9.7)	1.9 (0.90–3.9)	
		Total	90/1200	7.5 (6.0–9.0)		
						
*Blastocystis* spp.	Region	Datong	6/225	2.7 (0.6–4.8)	2.4 (0.6–9.8)	<0.001
		Jinzhong	5/428	1.2 (0.2–2.2)	1.0 (0.3–4.4)	
		Lvliang	3/267	1.1 (0.0–2.4)	1	
		Changzhi	23/102	22.6 (14.4–30.7)	19.8 (5.8–67.5)	
		Yuncheng	5/178	2.8 (0.4–5.2)	2.5 (0.6–10.8)	
	Average Altitude	>1000 m	37/1022	3.6 (2.5–4.8)	1.3 (0.5–3.5)	0.546
		<1000 m	5/178	2.8 (0.4–5.2)	1	
	Age	≥3 years	12/559	2.2 (1.0–3.4)	1	0.017
		<3 years	30/641	4.7 (3.1–6.3)	2.2 (1.1–4.4)	
	Sex	Male	6/179	3.4 (0.7–5.6)	1	0.907
		Female	36/1021	3.5 (2.4–4.7)	1.1 (1.4–2.5)	
		Total	42/1200	3.5 (2.5–4.5)		

**Table 2:** Occurrence of *Giardia duodenalis* genotypes (assemblages B and E) in this study in relation to different factors.

Factors	Categories	No. of Positive/Totality	Assemblage B (*n*)				Assemblage E (*n*)			
			No. of Positive	*bg*	*tpi*	*gdh*	No. of Positive	*bg*	*tpi*	*gdh*
Region	Jinzhong	35/428	30	30	0	0	5	5	0	0
	Lvliang	21/267	4	4	0	0	17	1	0	16
	Yuncheng	6/178	0	0	0	0	6	6	0	0
	Changzhi	25/102	1	1	0	0	24	20	7	16
	Datong	3/225	3	3	0	0	0	0	0	0
Age	<3 years	61/641	22	22	0	0	39	29	7	16
	≥3 years	29/559	16	16	0	0	13	3	0	16
Sex	Male	8/179	2	2	0	0	6	5	0	1
	Female	82/1021	36	36	0	0	46	27	7	31
Altitude	>1000 m	84/1022	38	38	0	0	46	26	7	32
	<1000 m	6/178	0	0	0	0	6	6	0	0
Total		90/1200	38	38	0	0	52	32	7	32

**Table 3:** Analysis of single nucleotide polymorphisms in *G. duodenalis* sequences at *bg* locus.

Sequences	Nucleotide at Position of Reference Sequence																No. of Sequences
**assemblage B**	**0**	**1**	**47**	**71**	**77**	**202**	**249**	**284**	**292**	**336**	**348**	**349**	**366**	**382**	**393**	**446**	
**LC184000-B** **Ⅲ**			A	C	G	A	T	A	C	T	T	C	A	T	A	A	
**PP754423**	**G**	**A**	**.**	**.**	**.**	**G**	**.**	**.**	**.**	**.**	**.**	**.**	**.**	**C**	**.**	**.**	**29**
**PP754424**	**G**	**A**	**.**	**.**	**.**	**G**	**.**	**.**	**.**	**.**	**.**	**.**	**.**	**C**	**.**	**G**	**1**
**PP754425**	**G**	**A**	**.**	**.**	**.**	**G**	**.**	**.**	**.**	**.**	**C**	**.**	**.**	**C**	**.**	**.**	**2**
**PP754427**	**G**	**A**	**.**	**.**	**.**	**.**	**.**	**.**	**.**	**G**	**.**	**.**	**T**	**.**	**.**	**.**	**1**
**PP754428**	**G**	**A**	**G**	**.**	**.**	**G**	**.**	**.**	**.**	**.**	**.**	**.**	**.**	**C**	**G**	**.**	**1**
**PP754429**	**G**	**A**	**.**	**T**	**.**	**G**	**.**	**.**	**.**	**.**	**.**	**.**	**.**	**C**	**.**	**.**	**1**
**PP754430**	**G**	**A**	**.**	**.**	**A**	**G**	**.**	**.**	**T**	**.**	**.**	**.**	**.**	**C**	**.**	**.**	**1**
**PP754431**	**G**	**A**	**.**	**.**	**.**	**G**	**G**	**.**	**.**	**.**	**.**	**.**	**.**	**C**	**.**	**.**	**1**
**PP754432**	**G**	**A**	**.**	**.**	**.**	**G**	**.**	**G**	**.**	**.**	**.**	**T**	**.**	**C**	**.**	**.**	**1**
																	
**assemblage E**	**29**	**416**															
**MK610387-E35**	**C**	**T**															
**PP754433-E35**	**.**	**.**															**27**
**PP754434-E41**	**G**	**.**															**1**
**PP754435-E1**	**.**	**C**															**4**

Nucleotide substitutions are in bold and in capital letters; dots indicate identical to the reference sequences.

**Table 4:** Distribution of *Blastocystis* spp. subtypes in black goats in Shanxi Province.

Factor		No. Positive/Tested	Subtype (*n*)
Region	Jinzhong	5/428	ST10 (3), ST5 (1), ST30 (1)
	Lvliang	3/267	ST10 (2), ST14 (1)
	Yuncheng	5/178	ST14 (5)
	Changzhi	23/102	ST10 (16), ST14 (6), ST5 (1)
	Datong	6/225	ST10 (5), ST14 (1)
Total		42/1200	ST10 (26), ST14 (13), ST5 (2), ST30 (1)

**Table 5:** Distribution of genotypes/subtypes of *G. duodenalis* and *Blastocystis* spp. in co-infected black goats in Shanxi Province.

Sequences	*G. duodenalis*			Blastcystis spp.
	*bg*	*tpi*	*gdh*	
**LC3, LC23, LC44, LC27**	E35	E33	E34	ST10
LC28	E35	-	E34	ST10
LC29	E35	-	-	ST10
ZY24	-	-	E34	ST14
**LC20**	E35	E33	E34	ST14
LC47	E35	-	-	ST14

Samples successfully amplified at three loci and forming a novel MLG-E12 are shown in bold.

## Data Availability

The data sets supporting the results of this article have been submitted to GenBank, and the accession number is shown in the article.

## Supplementary Materials

The following supporting information can be downloaded at: https://www.mdpi.com/article/10.3390/ani14121808/s1, Table S1: PCR primers used in this study; Table S2: Single nucleotide polymorphisms analysis of *G. duodenalis* sequences at *gdh* locus; Table S3: Single nucleotide polymorphisms analysis of *G. duodenalis* sequences at *tpi* locus; Table S4: Multilocus sequence genotyping of *G. duodenalis* based on the *tpi*, *gdh*, and *bg* loci; Table S5: Previously reported prevalence and assemblages of *G. duodenalis* in goats worldwide.
