# Supplementary material for: Prevalence and Genetic Characterization of Giardia duodenalis and Blastocystis spp. in Black Goats in Shanxi Province, North China: From a Public Health Perspective
Source: Animals (Basel). 2024 Jun 17;14(12):1808. doi: 10.3390/ani14121808 (PMC11201008; doi:10.3390/ani14121808)
Supplement: Supplementary file 1 [file animals-14-01808-s001.zip › Table S3.pdf]

Table S3. Single nucleotide polymorphisms analysis of *G. duodenalis* sequences at *tpi* locus.

Sequences	Nucleotide at position of reference sequence	No. of sequences
**assemblage E**	**298**	
**EU189333**	**G**	
**PP754421-E33**	**.**	**7**

Nucleotide substitutions are in bold and in capital letters, dots indicate identical to the references sequence.
