# Supplementary material for: Prevalence and Genetic Characterization of Giardia duodenalis and Blastocystis spp. in Black Goats in Shanxi Province, North China: From a Public Health Perspective
Source: Animals (Basel). 2024 Jun 17;14(12):1808. doi: 10.3390/ani14121808 (PMC11201008; doi:10.3390/ani14121808)
Supplement: Supplementary file 1 [file animals-14-01808-s001.zip › Table S4.pdf]

Table S4. Multilocus sequence genotyping of *G. duodenalis* based on the *tpi, gdh* and *bg* loci.

MLGs	Genotypes			NO. of samples
	bg	*gdh*	*tpi*	
MLG-E12	E35	E34	E33	**n=6**
-	E35	E34	-	n=5
-	E35	E53	-	n=1
-	E35	-	E33	n=1

NO. of samples successfully amplified at three loci and forming a MLG are in bold.
