# Supplementary material for: Prevalence and Genetic Characterization of Giardia duodenalis and Blastocystis spp. in Black Goats in Shanxi Province, North China: From a Public Health Perspective
Source: Animals (Basel). 2024 Jun 17;14(12):1808. doi: 10.3390/ani14121808 (PMC11201008; doi:10.3390/ani14121808)
Supplement: Supplementary file 1 [file animals-14-01808-s001.zip › Table S5.pdf]

Table S4: Previously reported prevalence and assemblage of *G. duodenalis* in goats worldwide.

Country	Region	No. of positive / tests	Prevalence (%)	*bg*	*tpi*	*gdh*	Years	References
China								
	Anhui	32/506	6.3%	-	E(n=23)	E(n=16)	*	[52]
	Gansu	6/13	46.2%	*	*	*	*	[49]
	Guangdong	56/226	24.8%	E(n=54) A+E(n=2)	-	-	2022	[29]
	Guizhou	5/53	9.4%	*	*	*	*	[49]
	Hainan	2/87	2.3%	*	*	*	*	[49]
	Heilongjiang	4/139	2.9%	-	*	-	2009-2011	[41]
	Henan	34/156	21.8%	*	*	*	*	[49]
	Inner Mongolia	21/561	3.7%	E(n=21)	E(n=5)	E(n=2) B(n=1)	2019-2021	[34]
	Jiangsu	36/120	30.0%	*	*	*	*	[49]
	Jilin	6/18	33.3%	*	*	*	*	[49]
	Liaoning	5/16	31.3%	*	*	*	*	[49]
	Qinghai	3/9	33.3%	*	*	*	*	[49]
	Shaanxi	33/202	16.3%	E(n=26) A(n=4)	E(n=12) A(n=4)	E(n=28) A(n=5)	2022-2023	[35]
	Shaanxi	93/1311	7.1%	E (n=56) A (n=4) B(n=3)	E (n=65) A (n=6)	E (n=23) A (n=2)	2014-2017	[40]
	Shaanxi	45/485	9.3%	-	E(n=76) A(n=4)	-	2014-2015	[51]
	Henan	35/144	24.3%					
	Sichuan	51/342	14.9%	E(n=21)	E(n=16)	E(n=10)	2017	[50]
	Tibet	0/260	0.0%	*	*	*	*	[42]
	Yunnan	38/907	4.2%	E(n=35) E(n=1) E(n=2)	E(n=10)	E(n=2) E(n=16) E(n=1)	2018	[30]
	Yunnan	16/336	4.8%	E (n=10) A (n=6)	E (n=5) A (n=7)	E (n=6) A (n=3)	2017	[19]
Ghana		35/285	12.3%	*	*	*	2014-2015	[48]
Greece		103/255	40.4%	*	*	*	*	[47]
India		70/207	33.8%	*	*	*	2016	[46]
Iran		15/94	16.0%	-	E(n=15)	-	2011-2012	[43]
Iran		5/100	5.0%	*	*	*	*	[38]

Malaysia	21/310	6.8%	*	*	*	*	[39]
Spain	39/315	12.4%	E(n=19)	E(n=31)	-	2006-2007	[44]
UK(farms in north-west England)	1/9	11.1%	*	*	*	2007-2008	[45]
