# Supplementary material for: Prevalence and Genetic Characterization of Giardia duodenalis and Blastocystis spp. in Black Goats in Shanxi Province, North China: From a Public Health Perspective
Source: Animals (Basel). 2024 Jun 17;14(12):1808. doi: 10.3390/ani14121808 (PMC11201008; doi:10.3390/ani14121808)
Supplement: Supplementary file 1 [file animals-14-01808-s001.zip › Table S1.pdf]

Table 1 PCR primers used in this study.

Species	Gene	Primer ID	Primer sequences (5'-3')	Annealing temperature	Fragment length
G. duodenalis	*bg*	BGF1	AAGCCCGACGACCTCACCCGCAGTGC	56 °C	
		BGR1	GAGGCCGCCCTGGATCTTCGAGACGAC		
		BGF2	GAACGAACGAGATCGAGGTCCG	58 °C	515 bp
		BGR2	CTCGACGAGCTTCGTGTT		
	gdh	GDH1	TTCCGTRTYCAGTACAACTC	55 °C	
		GDH2	ACCTCGTTCTGRGTGGCGCA		
		GDH3	ATGACYGAGCTYCAGAGGCACGT	58 °C	530 bp
		GDH4	GTGGCGCARGGCATGATGCA		
	tpi	ALF1	AAATIATGCCTGCTCGTCG	52 °C	
		ALR1	CAAACCTTITCCGCAAACC		
		ALF2	CCCTTCATCGGIGGTAACTT	60 °C	530 bp
		ALR2	GTGGCCACCACICCCGTGCC		
Blastocystis spp.	SSU rRNA	BhRDr-F1	GAGCTTTTTAACTGCAACAACG	65 °C	610 bp
		GR1	ATCTGGTTGATCCTGCCAGT		
